# Supplementary material for: Learning curve and surgical outcome of robotic assisted colorectal surgery with ERAS program
Source: Sci Rep. 2022 Nov 29;12:20566. doi: 10.1038/s41598-022-24665-w (PMC9709162; doi:10.1038/s41598-022-24665-w)
Supplement: Supplementary file 1 — Supplementary Information 1. [file 41598_2022_24665_MOESM1_ESM.zip › LSC Raw data-Ñ[▒K/131/H_ñw╝╨░O▒Kñσ.pdf]

正式摘要(電子簽章：000000223261964)		
W95 / W95 ☐英文標題(注意:無英文姓名-請補病人基本檔!)	☐【讀取】憑證	

臺中榮民總醫院

出院病歷摘要

轉入號 : 轉出號 :

[redacted]

(8)流水編號	
(9)入院日期	110/08/10 (2021) 直腸外科 W95 - 32
(10)轉科日期	
(11)出院日期	110/08/18 (2021) 住院天數計 8 日

診斷

- 入院 :1. diverticulitis.
- 出院 :1. Ascending colon diveticulitis s/p laparoscopic right hemicolectomy on 2021/08/11
  2. Uterine myoma s/p ATH at 20 y.
  3. Hypertension
  4. gall bladder stone related cholecystitis s/p OP at 8 y

主訴

- Abdominal pain for 6 months

病史

- This patient had history of :
  1. gall bladder stone related cholecystitis s/p OP at 8 y
  2. Uterine myoma s/p ATH at 20 y.
  3. Hypertension
  She had Abdominal pain for 6 months. She visited our OPD for help. She had abdomen CT on 2021/07/22.
  Which showed suspected diverticulitis.
  She was admitted for operation.

體檢發現

-
  - General appearance :
    - easy looking , conscious clear , without cardiopulmonary distress
    - Vital signs自動帶入date 體溫(BT) 脈搏(P) 呼吸(R) 血壓(BP) 身高(BH) 體重(BW) BMI
      20210810 36.4℃ 105次/分 18次/分 132/92mmHg 158.3cm 66.4kg 26.5
    - Vital signs: B.P. 132/9 mmHg , P.R. 105 /min , R.R. 18 /min , B.T. 36.4 ℃
  - HEENT :
    - Eyes: no anemic conjunctiva, no icteric sclera, normal EOM
    - ENT: no otorrhea, no rhinorrhea, no erythematous mucosa, no injected tonsil, no deformity
  - Neck: supple, freely movable, trachea midline,no deformity, no engorged jugular vein , no palpable thyroid or lymph node enlargement
  - Chest:
    - Inspection: Grossly normal, symmetric and fully expansion, no accessory muscle used, no operation scar, no spider angiomata, no gynecomastia
    - Palpation: no palpable mass or nodule, no subcutaneous emphysema, no tenderness
    - Ausculation: clear breathing sound
  - Heart:
    - Palpation: no thrill, no heave
    - Percussion: normal heart size
    - Ausculation: regular heart beat, normal S1, S2, no gallop, no murmur
  - Extremities: No deformity, full ranged of motion without limitation, no clubbing finger or nail cyanosis, no pitting edema, no ulcer or pigmentation, no obvious skin lesion,

- no varicose vein, no bone, joint or muscle tenderness

CRS CONDITION

- Karnofsky performance status: (100) Normal, no complaints, no evidence of disease
- PE of Abdomen:
- CEA: NIL ng/ml
- Imaging Studies:
- Colonoscope report: NIL
- Figures:
  1. colon & rectum

手術日期及方法

- 手術日期及方法20210811 1. Laparoscopic right hemicolectomy with side-to-side anastomosis, endo GIA 60-3.8mm*2+endo GIA 60-4.1mm*1 and Connel suture with 3-0 monocryl. re-enforced with 3-0 mersilk lembert suture.
  2. Vessel ligation: ileo-colic vessels.
  3. A Fr.12 penrose drain placed at Morrison's pouch.

住院治療經過

- She had operation on 2021/08/11. She recovered well, and discharged today.

合併症

一般檢查

- 累積報告 :

DATE	TIME	ALB	TP	BIL.T	BIL.D	ALKP	AST	ALT	LDH	AMY	LIPASE	NH3	Ferritin	UA
1100810	1659			0.67	0.12		42	52						
1100811	1154	3.8		0.7			30	42						

DATE	TIME	WBC	RBC	HGB	HCT	MCV	PLT	BLST	NEUT	BAND	SEG	LYM	MON	EOS	BAS
1100810	1658	6760		15.0					52.1			40.5	5.6	1.2	0.6
1100811	1156	9360		13.2			154		82.8			15.7	1.3	0.0	0.2

DATE	TIME	NA	K	CL	BUN	CREAT	eGFR	CA	Mg	P	CRP	ESR	PCT	D-Dimer	LACTATE
1100810	1659	141	3.8		10	0.77		9.2							
1100811	1154	140	3.3		7	0.61									

特殊檢查

病理報告

- 活體組織切片病理報告 手術日期：**110/08/11** 病理號：**S1024700**

診斷醫師:詹以吉 報告日期:110/08/17
Pathologic diagnosis:
Ascending colon, terminal ileum, and appendix, right hemicolectomy
--- Diverticulosis with chronic follicular diverticulitis. The appendix, ileocecal valve, bilateral cut ends and mesocolon are unremarkable.
Gross description:
The specimen consists of a segment of colon, 7.5 cm in length, and 6.5 cm in distal cut end circumference, and a segment of terminal ileum, 6 cm in length, and 3.5 cm in cut end circumference. An unremarkable appendix, 5 cm, is noted. There are three diverticula, up to 0.5 cm, noted at the mucosa of ascending colon, which are 1.8 cm from the ileocecal valve. The appendix, ileocecal valve, and both cut ends are free of tumor. Representative part for section: A) proximal cut end B) distal cut end C) appendic D) ileocecal valve
E-G) lesions.
#T-59420_2 #M-32700_2 0 1105 000000
#T-59420_2 #M-43000_2 0 1105 000000
#T-59200_2 #M-09183_2 0 1105 000000

其它

出院時情況

- 15.計劃性出院

出院指示

-

藥名	劑量	劑量單位	用法	頻次	數量	數量單位	現況	天數	單位
Ultracet tab	1	TAB	PO	Q6H	20	TAB	TH	5	天
Panho tab 40mg	1	TAB	PO	QDAC	5	TAB	TH	5	天
Econ tab 40mg	1	TAB	PO	QID	20	TAB	TH	5	天
Through tab	1	TAB	PO	QN	5	TAB	TH	5	天

出院計劃

主治醫師 2222H 林俊余 / 住院醫師 2340H 郭連雄

正式摘要(電子簽章：**000000223261964)**

W95 / W95

doctorRank:B
